# Comparisons of the survival time of patients with ovarian cancer adopting post-operative chemotherapy by use of paclitaxel combined with carboplatin or nedaplatin

**DOI:** 10.1186/s12957-016-0930-5

**Published:** 2016-06-24

**Authors:** Hongfei Gao, Lijun Yuan, Yimin Han

**Affiliations:** Department of Gynaecology, The Third Affiliated Hospital of Harbin Medical University, 150# Hapin Street, Xiangfang District, Harbin, 150040 Heilongjiang China; Social Medicine Department of Harbin Medical University, 157# Baojian Road, Nan’gang District, Harbin, 150081 Heilongjiang China

**Keywords:** Ovarian cancer, Carboplatin, Nedaplatin, Survival time, CA125

## Abstract

**Background:**

The current study aims to evaluate and compare the efficacy of post-operative chemotherapy using paclitaxel plus carboplatin or nedaplatin in patients with ovarian cancer, as well as the effects of different combinational therapies on the survival times of patients.

**Methods:**

Ninety-four patients were recruited for the study. These ovarian cancer patients were admitted into the Cancer Hospital Affiliated with Harbin Medical University for surgery from January 2008 to October 2009. They were divided into different groups according to their post-operative chemotherapy schemes: paclitaxel plus carboplatin (CBP group, *n* = 48) and paclitaxel plus nedaplatin (NDP group, *n* = 46). Variance analysis was used to compare the effects of different chemotherapy schemes and pathological types of ovarian cancer on the level of CA125 in serum at different treatment time points. Univariate and multivariate analyses were employed to evaluate the survival times of patients in different groups and pathological types and ages.

**Results:**

No significant differences were observed regarding the effects of various chemotherapy schemes (*P* = 0.561) and pathological types (*P* = 0.903) on the level of CA125 in serum of patients with ovarian cancer. However, the duration of chemotherapy had a profound impact on the level of CA125 in serum (*P* < 0.001). The survival times of patients was not affected by age (*P* = 0.101) and pathological type of ovarian cancer (*P* = 0.94) significantly. However, it was significantly affected by the chemotherapy scheme.

**Conclusions:**

Combined chemotherapy using carboplatin plus paclitaxel should be considered as the preferred treatment scheme for the initial treatment of ovarian cancer.

## Background

Ovarian cancer is the fifth most common cause of cancer death in women and is the most common cause of death by a gynecological tumor, which seriously threatens the health of women [1, 2]. Due to the insidious onset, rapid progression, easy intraperitoneal dissemination and metastasis in the early stage, and lack of effective early diagnosis of ovarian cancer, approximately 70 % patients are diagnosed in the advanced stages of the disease. So, cytoreductive surgery followed by adjuvant chemotherapy in the form of combined taxane and platinum remains the primary treatment [3, 4]. Chemotherapy using paclitaxel combined with platinum has been used to treat patients with ovarian cancer since the late twentieth century [4]. Due to less toxicity, easier administration, and higher patient tolerance of carboplatin compared with cisplatin, a chemotherapy regimen consisting of carboplatin plus paclitaxel has gradually replaced cisplatin plus paclitaxel, becoming the preferred treatment scheme in clinical practice for advanced ovarian cancer [5]. And, the overall 5-year survival rate of patients with ovarian cancer is approximately 20–30 % [6]. However, the resistance of carboplatin is still unresolved and this would lead to the recurrence of cancer indirectly.

Nedaplatin is a second-generation platinum-based chemotherapy drug with a similar mechanism of action to that of cisplatin, which can crosslink with DNA in a non-cell-cycle-specific manner. Its dissolution rate in plasma is ten times faster than that of cisplatin. Moreover, nedaplatin can be used without hydration. Furthermore, there is no cross resistance between nedaplatin and cisplatin or carboplatin [7]. Clinical studies showed that the effectiveness of nedaplatin plus paclitaxel is higher than that of carboplatin plus paclitaxel in the treatment of a malignant tumor. So, there is a tendency that nedaplatin would substitute carboplatin in clinic because the toxicity of nedaplatin is lower than that of carboplatin [8, 9]. Presently, both nedaplatin plus paclitaxel and carboplatin plus paclitaxel are feasible in the treatment of some malignant tumors [10]. However, there are few applications and limited evaluations of the efficacy of the clinical usage of nedaplatin plus paclitaxel in ovarian cancer, and the clinical efficacy and safety of paclitaxel plus nedaplatin in ovarian cancer still needs further validation.

Carbohydrate antigen 125 (CA125) is a kind of glycoprotein and a classical marker for early diagnosis and recurrence of ovarian cancer [11, 12]. The level of CA125 in serum is positively correlated with the degree of tumor malignancy and the possibility of recurrence. At the same time, in the same patient with ovarian cancer, rising and falling of CA125 value reflect the expanding and reduction of the lesions to a certain extent [13, 14].

In the current study, firstly, influences of different ovarian cancer pathologies, chemotherapy schemes, and treatment durations on the level of CA125 in serum were analyzed. Secondly, effects of paclitaxel plus carboplatin or nedaplatin on the survival times of patients with ovarian cancer were evaluated and compared. The results of this study would provide certain guidance for the clinical selection of chemotherapy schemes.

## Methods

### Inclusion criteria

From January 2008 to October 2009, patients with ovarian cancer admitted into the Gynaecology Department of the Cancer Hospital Affiliated to Harbin Medical University, Heilongjiang Province of China, who underwent ovarian cancer cytoreductive surgery and six courses of chemotherapy, and met the following criteria were recruited.

1. Patients with complete medical records and follow-up data from January 2013 to October 2014.
2. Patients diagnosed with primary and poorly differentiated epithelial ovarian adenocarcinoma in the post-operative pathological histological examination.
3. Phase IIIc patients who had received first surgery (cytoreductive surgery) in our hospital, with less than four lymph nodes metastasis, the maximum diameter of the residual tumor is smaller than 1 cm.
4. No history of chemotherapy before surgery.
5. The level of CA125 in serum had been measured before surgery and chemotherapy, and the level of CA125 in serum before surgery must be higher than the normal level (35 U/ml).
6. Patients received combination chemotherapy of paclitaxel plus carboplatin or nedaplatin after surgery.

### Grouping and the regimen of chemotherapy

Patients who met the inclusion criteria were divided into different groups based on their post-operative chemotherapy schemes: carboplatin plus paclitaxel (CBP group, 48 cases) and nedaplatin plus paclitaxel (NDP group, 46 cases). All patients in the two groups received combined chemotherapy. The detailed regimen in CBP group was paclitaxel (135–175 mg/m^2^) and carboplatin (area under the curve, five), administered intravenously and/or intraperitoneally every 3 weeks for six cycles. Similarly, the detailed regimen in NDP group was paclitaxel (135–175 mg/m^2^) and nedaplatin (80–100 mg/m^2^), administered intravenously and/or intraperitoneally every 3 weeks for six cycles.

### Determination of the level of CA125 in serum

For each patient, 3 ml of fasting venous blood was collected in a sterile vacuum blood tube via the conventional method. The collected blood samples were kept at room temperature for 0.5 h and then centrifuged at 3000 rpm/min for 10 min to obtain the serum samples, which were stored at −80 °C until analysis. The current study employed the electrochemical luminescence method to measure the level of CA125 in serum by using a chemiluminescence immunoassay analyzer and accompanied reagents purchased from Roche, Switzerland. All measurements were conducted strictly according to the standard operating procedures of the instruments and manufacturer’s instructions. The level of CA125 in serum was considered to be positive when it is higher than 35 U/ml.

### Statistical analysis

EpiData 3.02 software was used for data entry, and all statistical analyses were performed using SPSS 17.0. Some results are presented as means ± standard deviation (SD). Student’s *t* test and the chi-square test were used to compare the ages and the pathological type of ovarian cancer between the two groups. Repeated analysis of variance was employed to compare the effects of different chemotherapy schemes and the pathological type of ovarian cancer on the level of CA125 in serum at different time points. Furthermore, Kaplan–Meier (KM) univariate survival analysis was used to compare the effects of different chemotherapy schemes and the pathological types of ovarian cancer on the survival times of patients. In addition, Cox regression analysis was employed to assess the influences of various factors on the survival times of patients with ovarian cancer. All tests were two-tailed, and a *P* value <0.05 was considered to be statistically significant.

## Results

### Comparisons of age and pathological types of patients between the two groups

Among the 94 patients, the youngest was 26 years of age and the oldest was 80 years of age. No statistically significant differences in average age and pathological types were seen (*P*_age_ = 0.558, *P*_pathological types_ = 0.763), indicating that they were balanced and comparable between the two groups. Specific data are listed in Table 1.

**Table 1:** Comparisons of ages and pathological types of patients between two groups

	CBP (*n* = 48)	NDP (*n* = 46)	Statistics	*P* value
Age	50.75 ± 8.566	51.87 ± 9.854	t = 0.589	0.558
Pathological type			$\chi$ ^2^ = 0.091	0.763
Poorly differentiated adenocarcinoma (30)	16	14		
Papillary serous adenocarcinoma (64)	32	32		

### The effects of different chemotherapy schemes, durations, and pathological types on the level of CA125 in serum

**Fig. 1:**
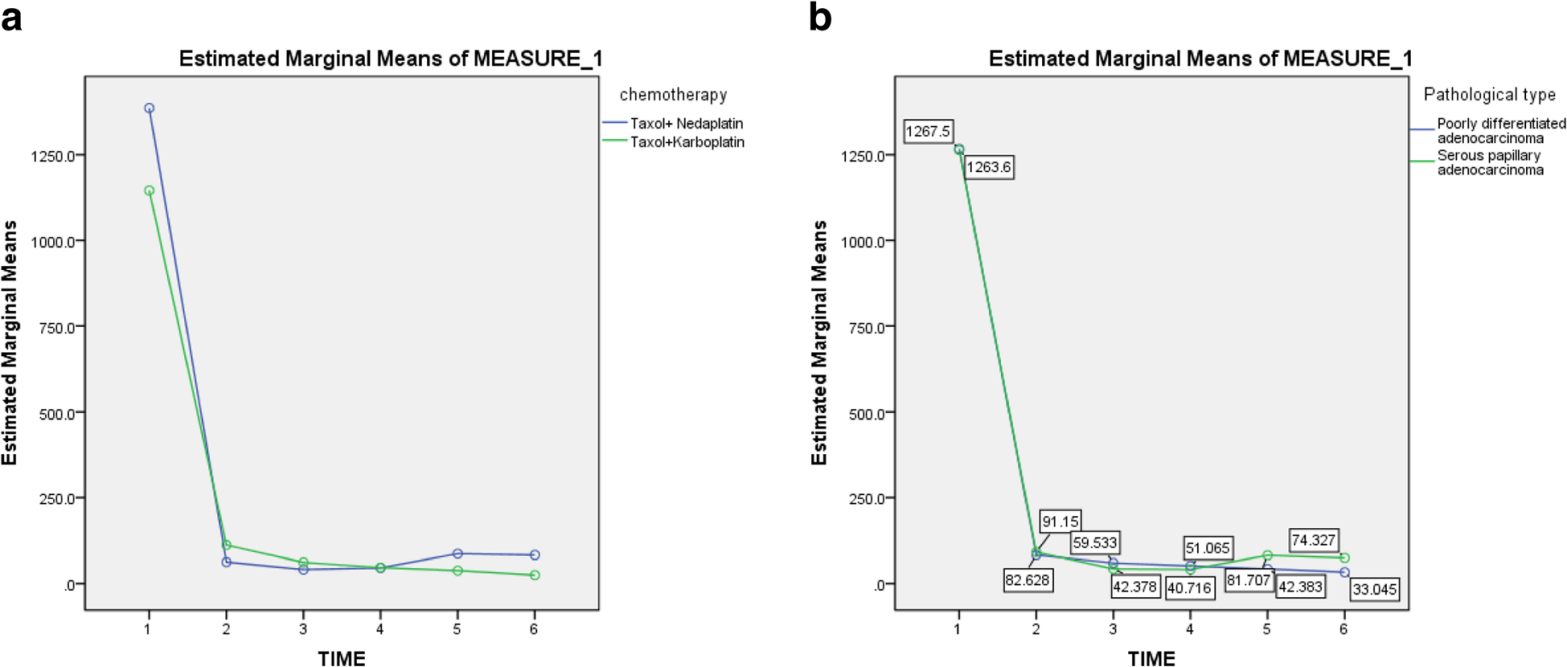
The level of CA125 in serum of different groups and pathological types. **a** The level of CA125 in serum of patients in different groups. **b** The level of CA125 in serum of patients with different pathological types

There was no significant difference between the level of CA125 in serum of patients in the different groups and between different pathological types (F_chemotherapy scheme_ = 0.342, *P* = 0.561; F _pathological types_ = 0.015, *P* = 0.903). The level of CA125 in serum of the different groups and pathological types are shown in Fig. 1. However, with the duration of chemotherapy increased, the level of CA125 in serum significantly decreased (*F* = 41.395, *P* < 0.001).

### The results of KM univariate survival analysis

**Fig. 2:**
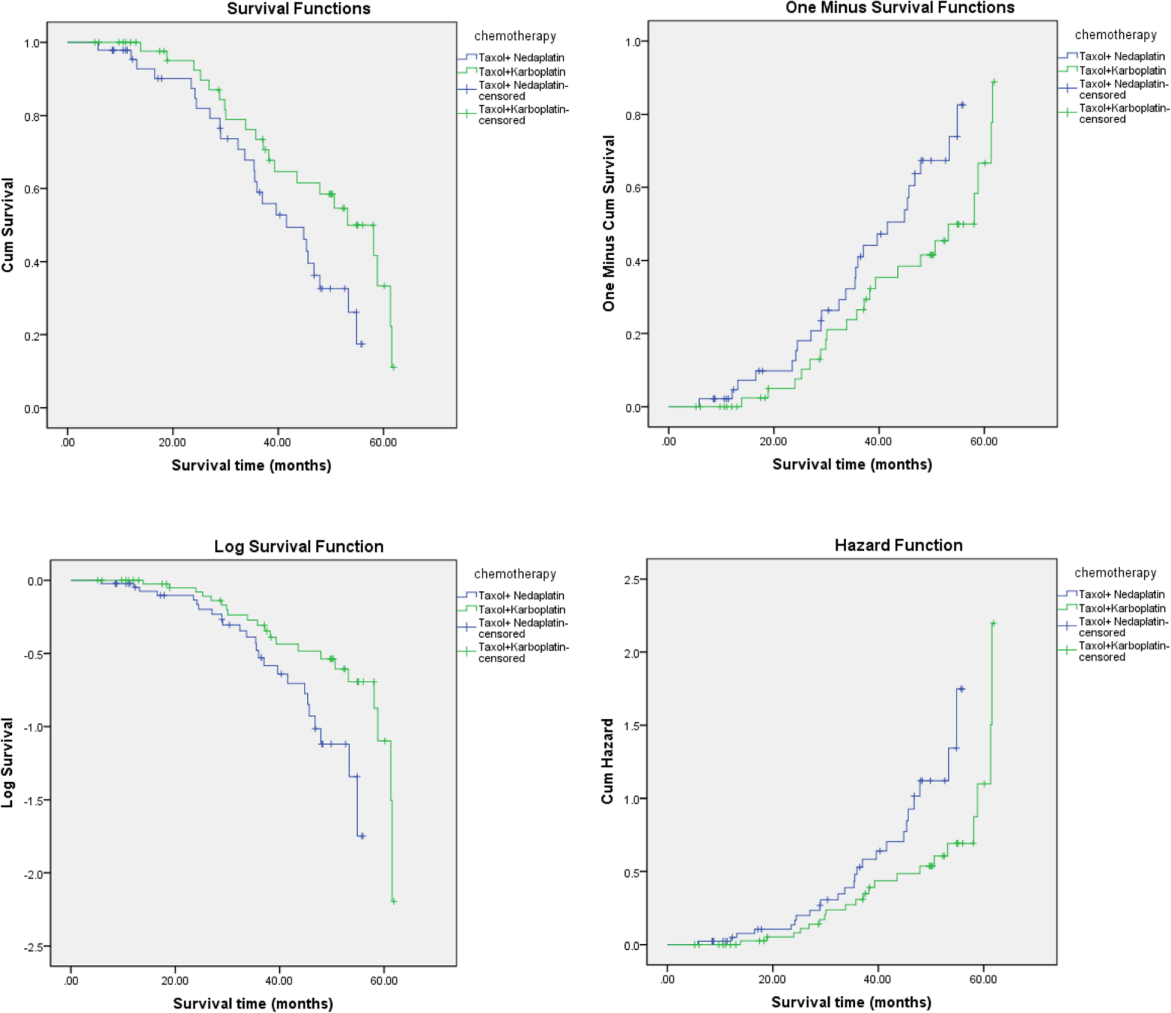
The influence of different chemotherapy schemes on the survival time of patients

**Fig. 3:**
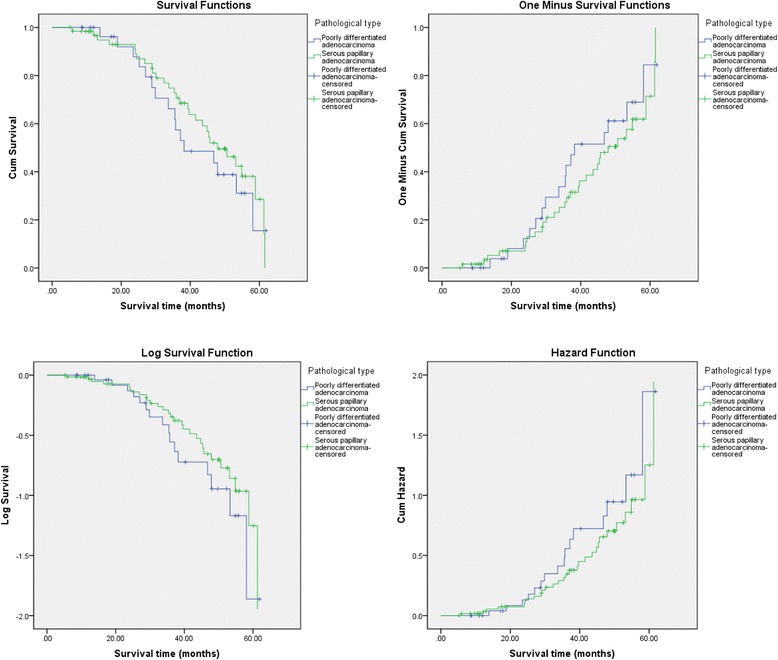
The influence of different pathological types on the survival time of patients

The results demonstrated that there was a significant difference in survival times between patients of different chemotherapy schemes ($\chi$^2^ = 4.801, *P* = 0.028). Namely, the survival time of patients in the CBP group was significantly longer than that in the NDP group (Fig. 2). However, there was no significant difference in survival times of patients with different pathological types ($\chi$^2^ = 0.447, *P* = 0.504, Fig. 3).

### The results of Cox multivariate survival analysis

The results of Cox regression analysis showed that the age and pathological types of patients did not affect their survival times (*P*_age_ = 0.101, *P*_pathological types_ = 0.94), while different chemotherapy schemes significantly affected the survival times of patients (*P* = 0.029). The results also indicated that those patients in the CBP group had a lower risk to die, with a value of 0.501 (95 % CI: 0.269–0.932) when compared to those in the NDP group, signifying that the survival time of patients in the CBP group was longer than those in the NDP group. The data is presented in Table 2.

**Table 2:** The results of Cox multivariate survival analysis

Factors	Wald	*P*	Exp (B)	95 % CI	
				Lower	Upper
Age	2.694	0.101	1.03	0.994	1.068
Chemotherapy schemes	4.756	0.029	0.501	0.269	0.932
Pathological type	0.006	0.94	1.025	0.535	1.963

## Discussion

CA125, found in 1981, is an inhomogeneous, highly molecular mucin-like glucoprotein. In patients with effective surgical treatment and chemotherapy, the level of CA125 in serum decreases quickly. In cases of recurrence and drug resistance, the level of CA125 in serum elevates prior to the clinical symptoms, indicating that CA125 is an early marker for recurrence and drug resistance. In the current study, post-operative chemotherapy lowered the level of CA125 in serum of patients in both the groups; especially with a longer duration of post-operative chemotherapy, the level of CA125 in serum significantly decreased. The results suggested that the duration of chemotherapy is the main factor affecting the level of CA125 in serum of patients. This result is consistent with previous studies. Li et al. found that the recurrence rate of ovarian cancer closely correlated with the type of post-operative chemotherapy; at the same time, completion of post-operative chemotherapy was a significant factor influencing the prognosis. And, they found that the recurrence rate of patients who completed post-operative chemotherapy (27.6 %) was significantly lower than that of the patients who did not (47.3 %) [15].

Carboplatin in combination with paclitaxel has been broadly accepted as first-line chemotherapy for advanced epithelial ovarian cancer for many years [16, 17]. During the initial design of this study, researchers hypothesized that the survival time of patients in the NDP group would be longer than those in the CBP group because less side effects and higher patient tolerance of nedaplatin. And, some literatures partly support this hypothesis [18, 19]. Unexpectedly, although there was no significant difference in the level of CA125 in serum between patients receiving different chemotherapy schemes, the survival time of patients in the CBP group was longer than those in the NDP group. In addition, the price of nedaplatin is about 2.5 times that of carboplatin and this would increase the economic burden of patients preferring less toxicity. So, according to the results of this study, carboplatin plus paclitaxel should be continued in the treatment of ovarian cancer.

Pathological type of ovarian cancer affects the degree of malignancy and, subsequently, different rates of recurrence. Previous multivariate analysis showed that the pathological type was an independent risk factor for the recurrence of ovarian cancer. Serous carcinoma usually recurred, while mucinous carcinoma had better prognosis [20]. Another research reported that, relative to serous papillary cystadenocarcinoma and poorly differentiated carcinoma, papillary mucinous cystadenocarcinoma has better prognosis [21]. Our study also proved this view indirectly. In this study, we only focused on two pathological types of ovarian cancer with poor prognosis, including poorly differentiated carcinoma and serous papillary cystadenocarcinoma. And, there were no significant differences in the level of CA125 in serum and the survival time among patients with different pathological types at different time points.

## Conclusions

So, in this study, a chemotherapy scheme was the main factor to affect the survival time of patients with ovarian cancer. And, combined chemotherapy using carboplatin plus paclitaxel should be considered as the preferred treatment scheme for the initial treatment of ovarian cancer.

## Abbreviations

CA125, carbohydrate antigen 125; CBP, carboplatin plus paclitaxel; CI, confidence interval; KM, Kaplan–Meier; NDP, nedaplatin plus paclitaxel; SD, standard deviation
